# Hepatoprotective Activity of the Fruits of *Eleutherococcus senticosus* in Acetaminophen-Induced Liver Injury in Mice and Their Chemical Composition

**DOI:** 10.3390/nu17213456

**Published:** 2025-11-01

**Authors:** Filip Graczyk, Krystian Krolik, Dorota Gawenda-Kempczyńska, Magdalena Wójciak, Ireneusz Sowa, Dorota Sulejczak

**Affiliations:** 1 Department of Pharmaceutical Botany and Pharmacognosy, Ludwik Rydygier Collegium Medicum, Nicolaus Copernicus University, 9 Marie Curie-Skłodowska Street, 85-094 Bydgoszcz, Polanddgawenda@cm.umk.pl (D.G.-K.); 2 Department of Analytical Chemistry, Medical University of Lublin, Chodźki 4a, 20-093 Lublin, Poland; magdalena.wojciak@umlub.edu.pl (M.W.);; 3 Department of Experimental Pharmacology, Mossakowski Medical Research Institute, Polish Academy of Sciences, Pawinskiego 5, 02-106 Warsaw, Poland; dsulejczak@imdik.pan.pl

**Keywords:** *Eleutherococcus senticosus*, adaptogens, hepatoprotection, hepatotoxicity, mice

## Abstract

**Background/Objectives:** *Eleutherococcus senticosus* (Siberian ginseng) is an adaptogenic plant widely recognized for its antioxidant and immunomodulatory properties; however its hepatoprotective potential properties are unexplored. This study aimed to evaluate whether the fruit extract of *E. senticosus* contains chemical constituents with hepatoprotective effects in a paracetamol-induced liver injury model in mice. **Methods:** Female BALB/c mice were randomized into five groups: control, paracetamol (300 mg/kg, IP), *E. senticosus* extract (750 or 1500 mg/kg, PO) + paracetamol, and silymarin (50 mg/kg) + paracetamol. Extracts were administered for seven days before paracetamol challenge. Biochemical markers (ALT, AST, urea, creatinine, protein, albumin) and hematological parameters were assessed, and organs were subjected to histopathological examination. Phytochemical characterization of the extract was performed using UHPLC-DAD-MS and ICP-OES. **Results:** The 750 mg/kg dose of *E. senticosus* extract maintained ALT, AST, urea, and creatinine levels close to control values, while the higher dose (1500 mg/kg) was less effective and showed an increase in serum urea. Both extract doses and silymarin attenuated creatinine elevation induced by paracetamol. No histopathological changes were detected in the kidneys or brains of treated animals. Phytochemical analysis revealed high contents of phenolic acids (chlorogenic and dicaffeoylquinic acids), flavonoids, amino acids, and essential minerals. **Conclusions:** *E. senticosus* fruit extract demonstrated a hepatoprotective effect at an optimal dose (750 mg/kg), indicating a potential dose-dependent effect. The absence of histopathological alterations in key organs supports the fruit extract’s safety.

## 1. Introduction

*Eleutherococcus senticosus* (Rupr. et Maxim.) Maxim. (ES), commonly known as Siberian ginseng, has been gaining interest for decades as a natural remedy supporting immunity and the body’s adaptation to stress. Its action as an adaptogen and immunostimulant has been used in traditional Chinese and Russian medicine for centuries, where the roots were used as a tonic to strengthen, prolong life, and improve overall well-being [1,2]. In Europe, research on this plant intensified in the second half of the 20th century, and *E. senticosus* is now widely used as an ingredient in supplements that support immunity and improve physical and mental performance. Its rich phytochemical composition includes eleutherosides, flavonoids, lignans, and phenolic acids, which are believed to have anti-inflammatory, antioxidant, and immunomodulatory properties [3].

The fruit of *Eleutherococcus senticosus* is a berry that can be soaked in water or processed into products such as fruit wine and fruit vinegar. These preparations are valued for their contribution to daily health care [4]. Recent research has highlighted the nutritional and functional potential of *E. senticosus* fruits. Besides the well-characterized eleutherosides, the fruits are a natural source of dietary polyphenols (including lignans (sesamin, syringaresinol), phenolic acids (chlorogenic acid, caffeic acid), polysaccharides, coumarins, and flavonoids (quercetin, kaempferol)), which contribute to their antioxidant activity and may provide protection against oxidative stress associated with liver injury [5,6,7]. The fruits also contain high levels of minerals, including calcium (3730–4495 mg/kg), magnesium (1430–1540 mg/kg), iron (35.4–53 mg/kg), manganese (75.2–88.3 mg/kg), zinc (18.9–41.0 mg/kg), copper (3.34–13 mg/kg), and selenium (0.19–0.61 mg/kg) [8]. Notably, significant amounts of myo-inositol and D-mannitol were detected [8,9,10]. Compositional analyses of ES fruits have shown that, alongside polyphenols, they also contain fatty acids and tocopherols. This combination not only supports their antioxidant properties but also expands their potential applications in pharma-nutrition [11]. Among the best-known active compounds obtained from the ES extracts, eleutheroside B (syringin) and eleutheroside E (syringaresinol) are the best known, and have been shown to influence enzymatic activity and cell membrane stability. Furthermore, studies on plant extracts containing these compounds suggest their ability to regulate ion transport in epithelial cells and alleviate oxidative stress and inflammation [12,13].

*Eleutherococcus senticosus* has wide-raging spectrum of health-promoting properties, including potential hepatoprotective effects. Its beneficial results on the liver have not yet been as extensively studied as other hepatoprotective herbs, such as *Silybum marianum* L. (milk thistle), which contains silymarin, a flavonolignan witch gargantuan hepatoprotective properties. It has been extensively studied in animal models, such as mice, to protect the liver from a variety of damages, including those induced by toxins such as acetaminophen (paracetamol) [14,15,16,17].

In our study, we used intractum, an extract obtained by macerating fresh plant material in water-ethanol mixture. Such a method of preparation ensures the preservation of a broader spectrum of active compounds, including volatile and easily degradable substances, which may be lost during drying. This allows the extract to better reflect the natural composition and biological activity of the fresh plant. To date, there is a lack of studies conducted to confirm the *E. senticosus* fruit extract’s hepatoprotective properties. Currently, available research suggests certain mechanisms that may support liver protection. In vitro studies have shown that *E. senticosus* fruit extract contains chemicals that may have beneficial effects on liver cells. Furthermore, the extract exerted an immunomodulatory effect by proliferating peripheral blood leukocytes, reducing their susceptibility to VSV infection [9]. In addition, compounds found in both the fruits and roots of *E. senticosus* have been shown to influence the activity of enzymes such as hyaluronidase, suggesting their potential role in managing conditions associated with inflammation [18]. *E. senticosus* has also been reported to decrease cardiovascular responses to stress, supporting its role in stress adaptation [19]. Moreover, leaf extracts of *E. senticosus* have shown antidiabetic activity and improvement of lipid metabolism, suggesting potential applications in diabetes management [20].

Therefore, the aim of the present study was to determine whether the fruit extract of *Eleutherococcus senticosus* exhibits hepatoprotective properties in a mouse model of paracetamol-induced liver damage. And finally, whether the fruits can be used as food ingredients.

## 2. Results and Discussion

In preclinical studies, mice are often used as an animal model to study the hepatotoxicity of paracetamol. Typically, they are given appropriate doses of paracetamol to induce toxic effects similar to those observed in humans. Paracetamol is metabolized in the liver, primarily by the cytochrome P450 enzyme system, particularly the CYP2E1 isoform. A small portion of this metabolism leads to the formation of a toxic metabolite, NAPQI (*N*-acetyl-p-benzoquinoneimine). At high concentrations, NAPQI binds to proteins in liver cells (hepatocytes), causing damage and necrosis. This leads to acute liver damage. Accumulation of NAPQI also leads to oxidative stress, which further damages hepatocytes and enhances inflammatory processes. Over time, hepatocyte damage leads to an inflammatory response and the development of liver necrosis, which is a clinical manifestation of hepatotoxicity [14,17,21]. Silymarin, a flavonolignan extracted from milk thistle (*Silybum marianum*), is known for its strong hepatoprotective properties. Its effectiveness has been extensively documented in animal models, including mice, where it has been shown to protect the liver from damage caused by toxins such as acetaminophen. The action of silymarin is based primarily on its antioxidant properties–this compound effectively neutralizes free radicals and reduces oxidative stress, which plays a key role in the pathogenesis of liver damage, especially in the presence of toxic metabolites such as NAPQI. Additionally, silymarin supports the restoration of glutathione, which is essential for detoxification processes in the liver. Through these mechanisms, it may limit liver damage even in the event of a paracetamol overdose [15,16,21]. The roots of *Eleutherococcus senticosus*, known as an adaptogen, exhibit a wide range of beneficial health properties, including potential liver protection. However, a hepatoprotective potential of the fruits has not yet been studied as thoroughly as that of the roots or silymarin. Available data suggest mechanisms that may support liver protection.

The study was performed in mice, which are commonly used preclinical models due to physiological similarities to humans. While direct extrapolation to humans should be performed with caution, the observed protective effects of *Eleutherococcus senticosus* extract provide valuable insights into its potential activity. These findings may help guide further research in other models and inform possible relevance to human health.

### 2.1. Evaluation of the Hepatoprotective Effect of the Eleutherococcus senticosus Fruit Extract in a Model of Liver Damage with Paracetamol

**Figure 1:**
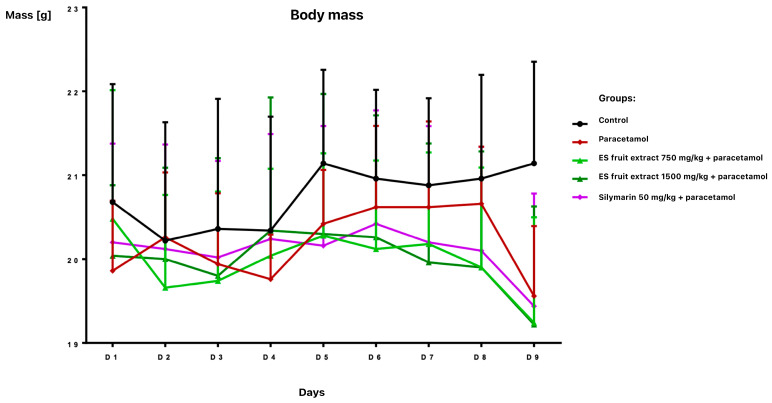
Body weight [g] of mice receiving the test preparations.

The study provided significant new evidence for the hepatoprotective properties of *Eleutherococcus senticosus* fruit extract in a mouse model of paracetamol-induced liver damage. The effects of *Eleutherococcus senticosus* fruit extract (750 and 1500 mg/kg) and silymarin (50 mg/kg) on paracetamol-induced alterations in hematological and biochemical parameters in mice were evaluated. Oral administrations were well tolerated, with no adverse effects observed in any group during the 7-day pre-treatment period. Body weight remained stable until day 8 (Figure 1).

**Figure 2:**
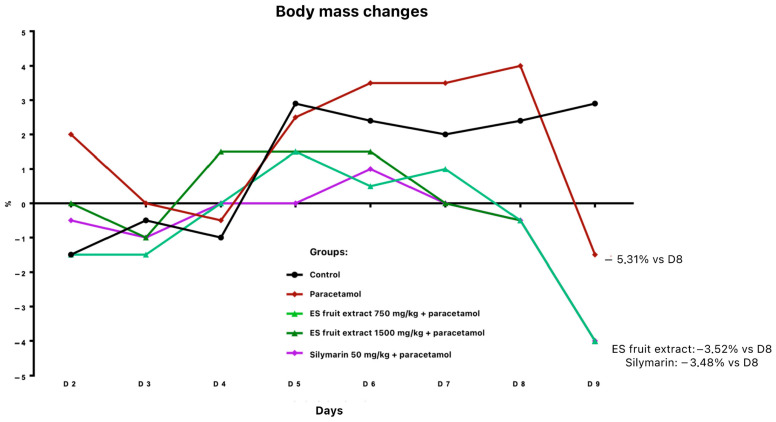
Changes in body weight [%] receiving the test preparations.

Twenty-four hours after paracetamol administration, a body weight reduction was noted: 3.5% in groups pre-treated with fruit extract or silymarin, and 5.3% in the paracetamol-only group (relative to day 8) (Figure 2). All paracetamol-treated animals exhibited ruffled fur.

Total organ weights suggested a trend towards decreased liver and spleen weights in pre-treated groups; However, when organ weights were adjusted relative to body weight, no statistically significant differences were observed. Lung weights were slightly higher in paracetamol-treated mice (Table 1). This suggests that preventive administration of an adaptogen or silymarin partially alleviates the acute metabolic stress induced by paracetamol overdose, which may be due to maintaining better water-electrolyte homeostasis and general condition of the mice.

**Figure 3:**
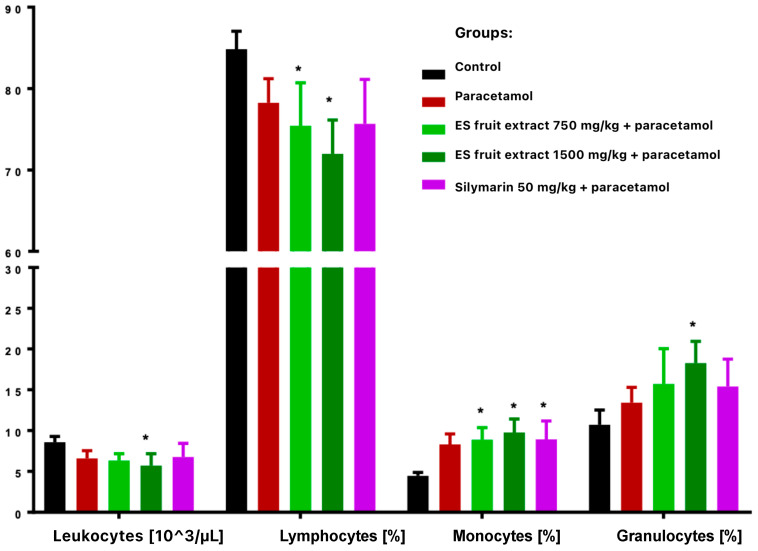
White blood cells smear: leukocytes [10^3^/µL], lymphocytes [%], monocytes [%], granulocytes [%]. * *p* < 0.05 nonparametric Kruskal–Wallis test, comparison vs. control.

**Figure 4:**
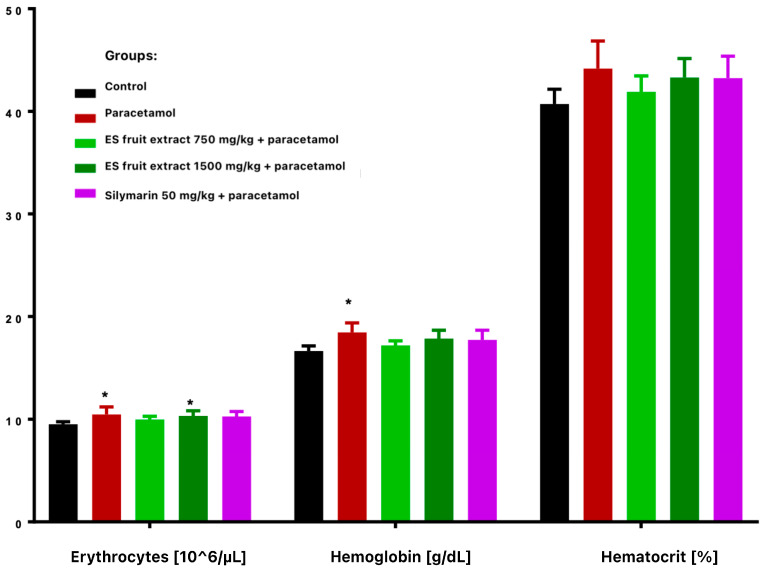
Red blood cells parameters: erythrocytes [10^6^/µL], hemoglobin [g/dL], hematocrit [%]. * *p* < 0.05 nonparametric Kruskal–Wallis test, comparison vs. control.

Paracetamol treatment reduced total leukocyte count and altered leukocyte distribution, with decreased lymphocytes and increased monocytes and granulocytes (Figure 3). These changes were more vivid and statistically significant in extract-treated groups compared with controls. Paracetamol alone significantly increased erythrocyte count and hemoglobin concentration (Figure 4), while hematocrit increase was not significant.

**Figure 5:**
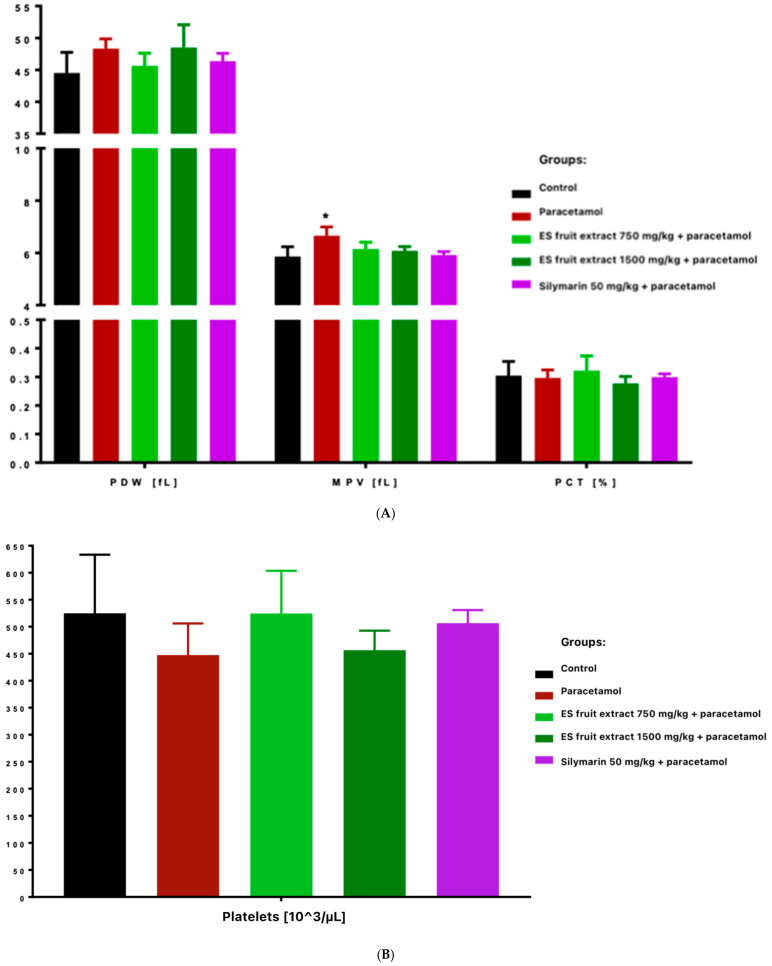
Platelet parameters: platelet distribution width [fL], mean platelet volume [fL], plateletcrit [%] (**A**) and platelets [10^3^/µL] (**B**). * *p* < 0.05 nonparametric Kruskal–Wallis test, comparison vs. control.

Platelet count decreased in paracetamol and high-dose ES fruit extract (1500 mg/kg) groups, accompanied by increased platelet anisocytosis (PDW) and mean platelet volume (MPV), and reduced plateletcrit (PCT) (Figure 5). Those results correspond to an acute stress and inflammatory reaction-paracetamol, especially in high doses, may cause the displacement of leukocytes (especially lymphocytes) into damaged tissues and the mobilization of neutrophils. In other words, the adaptogen did not prevent the decrease in leukocytes after paracetamol and even seemed to enhance it (these differences were statistically significant compared to the control group).

The most interesting results concern platelet parameters. The overdose of the paracetamol caused a significant reduction in platelet count. This was accompanied by an increase in platelet anisocytosis (PDW) and mean platelet volume (MPV) with a decrease in platelet permeability (PCT). This finding suggests increased platelet destruction or consumption (e.g., via microthrombi in the damaged liver) and simultaneous compensatory production of young, larger platelets by the bone marrow. Importantly, in the group protected with the lower dose of ES extract (750 mg/kg), no significant decrease in platelet count was observed, whereas with the high dose of ES extract (1500 mg/kg), thrombocytopenia was evident and similar to that in the paracetamol group. This indicates that the lower dose of the extract could protect megakaryocytes or inhibit platelet destruction (e.g., by limiting liver endothelial damage and microthrombi formation), whereas the higher dose did not produce this effect. Moreover, thrombocytopenia was even more pronounced in the high-dose ES group. It is possible that the surplus of some compounds in the high-dose ES had an adverse effect on platelet function or the coagulation system.

**Figure 6:**
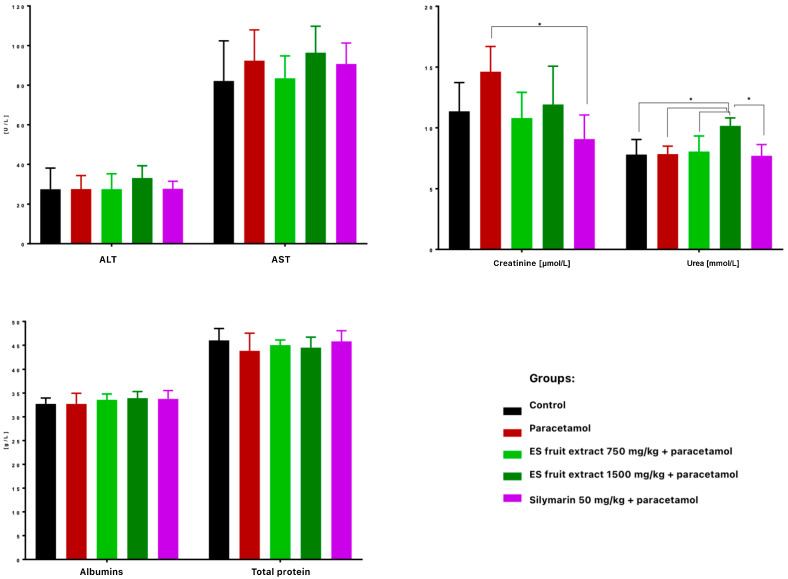
Biochemical parameters in mouse serum after administration of the tested substances: AST [U/L], ALT [U/L], creatine [µmol/L], urea [mmol/L], albumins [g/L], total protein content [g/L]. * *p* < 0.05 nonparametric Kruskal–Wallis test, comparison vs. control.

When it comes to the biochemical parameters, serum ALT and AST activities were not significantly elevated 24 h after paracetamol administration, suggesting no hepatotoxicity (Figure 6). Nonetheless, *Eleutherococcus senticosus* fruit extract at 750 mg/kg maintained ALT, AST, creatinine, and urea levels close to control values, whereas the 1500 mg/kg dose increased ALT (non-significant) and significantly raised serum urea. No significant changes were observed in total protein or albumin concentrations. Similarly, in a study on rats, a hepatoprotective effect was observed after administration of the root and rhizome extract. Lower doses (100, 300, and 500 mg/kg) led to a reduction in elevated AST and ALT levels induced by acetaminophen administration. Interestingly, in this case as well, a high dose of the extract (1000 mg/kg) caused an increase in the tested parameters. This confirms that the hepatoprotective effect of *Eleutherococcus senticosus* extract may be dose-dependent, and administration of excessively high doses may result in an opposite effect [22].

Much more visible changes were observed in renal function indices. High-dose paracetamol can also cause kidney damage (e.g., acute renal tubular damage), as reflected by increased serum creatinine levels (Figure 6). A significant increase in creatinine levels was observed in the paracetamol group compared to the control group. Interestingly, the ES extract supplementation mitigated this increase–creatinine was lower at both doses than with paracetamol alone, with the effect at the 750 mg/kg dose (with levels similar to the control group) and slightly weaker at the 1500 mg/kg dose. Silymarin significantly prevented the increase in creatinine, so this could suggest that ES, like silymarin, may have a nephroprotective effect in the context of paracetamol poisoning.

Although there is limited data on the nephroprotective effects of *Eleutherococcus senticosus* extract, such an effect has previously been confirmed another adaptogenic plant-*Panax ginseng*. In a study on rat model, in which administration of hydroxyurea led to elevated levels of creatinine and urea, treatment with the drug in combination with *Panax ginseng* extract resulted in a reduction in these parameters (in the case of urea, even to levels lower than the control) [23]. Both *Panax ginseng* and *Eleutherococcus senticosus* are plants with adaptogenic properties. It is possible that ES extracts may also have a protective effect on the kidneys. However, further research is necessary to confirm this hypothesis.

Despite the absence of marked ALT/AST elevation after paracetamol (300 mg/kg, IP), extract at 750 mg/kg exhibited a protective trend on hepatic enzyme activity and renal function markers, as well as favorable effects on platelet parameters. The absence of significant elevation of ALT, AST, urea, and creatinine in the 750 mg/kg group compared to control indicates that the extract effectively prevented paracetamol-induced hepatotoxicity. Conversely, the higher ES fruit extract dose (1500 mg/kg) showed less benefit and some adverse trends (e.g., elevated urea). Both extract and silymarin potentiated the paracetamol-induced reduction in leukocytes and shift in leukocyte subpopulations.

**Figure 7:**
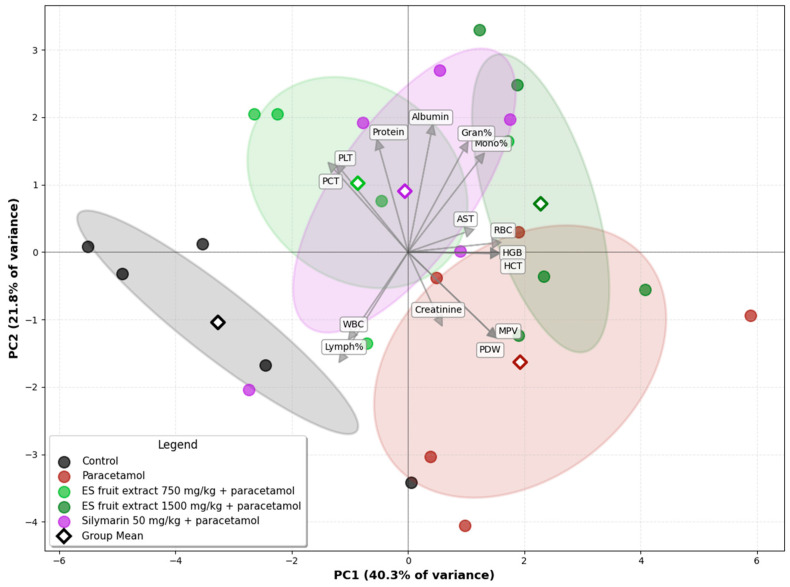
Principal Component Analysis (PCA) of the experimental groups based on biochemical and hematological parameters. Ellipses: 95% confidence interval for the group mean.

The Principal Component Analysis (PCA) of the experimental groups, based on biochemical and hematological parameters, illustrates the relationships among them (Figure 7). A clear separation is evident on the biplot between the control group (left side) and the paracetamol-only group (right side), along the PC1 axis. This axis accounts for the largest proportion of the data variance (40.3%) and can be interpreted as the axis of paracetamol-induced injury. The second principal component (PC2) explains 21.8% of the data variance. The position of the paracetamol group is associated with the vectors for variables such as creatinine and AST, as well as platelet parameters indicative of an inflammatory state (MPV, PDW), which graphically confirms the multi-organ toxicity of paracetamol. The group treated with the *E. senticosus* extract at a dose of 750 mg/kg (+paracetamol) is shifted towards the control group, and its group mean is located in close proximity to the mean of the reference silymarin group. The position of the ES fruit extract 750 mg/kg + paracetamol group between the Control and Paracetamol groups along the PC1 axis suggests that the lower extract dose partially mitigates the negative effects of paracetamol. Furthermore, this group’s confidence ellipse overlaps with that of the Silymarin 50 mg/kg + paracetamol group, suggesting that the overall effect of the 750 mg/kg ES fruit extract is similar to that of silymarin. The position of the ES fruit extract 1500 mg/kg + paracetamol group confirms that the higher dose of the extract did not exhibit a protective effect and, in fact, may have intensified the negative effects.

**Figure 8:**
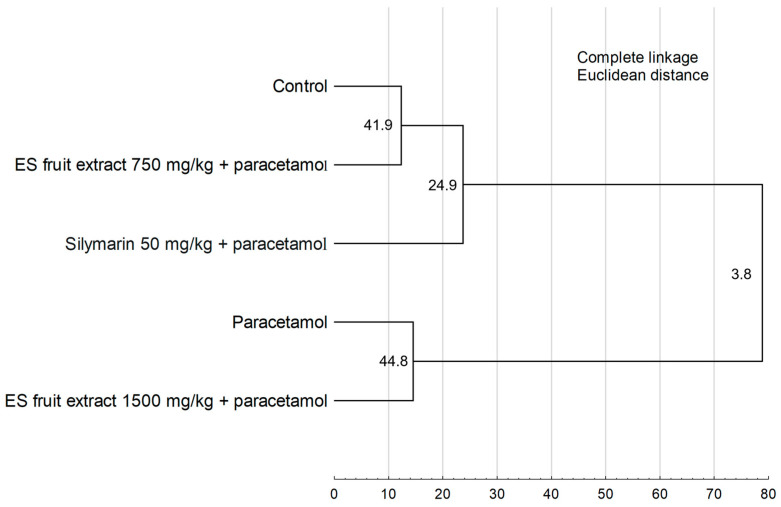
Hierarchical cluster analysis of the experimental groups. Numbers at the nodes represent bootstrap support values (from 1000 replicates). Cophenetic correlation coefficient = 0.9548.

The results of the Hierarchical Cluster Analysis (Figure 8) confirm the findings from the PCA, revealing two main, distinctly separated clusters. One cluster group, the control group, the group treated with the 750 mg/kg ES extract, and the silymarin group. The second cluster consists of the paracetamol-only group and the group treated with the higher 1500 mg/kg ES extract dose. The close similarity between these latter two groups demonstrates that the 1500 mg/kg dose failed to provide a protective effect.

**Figure 9:**
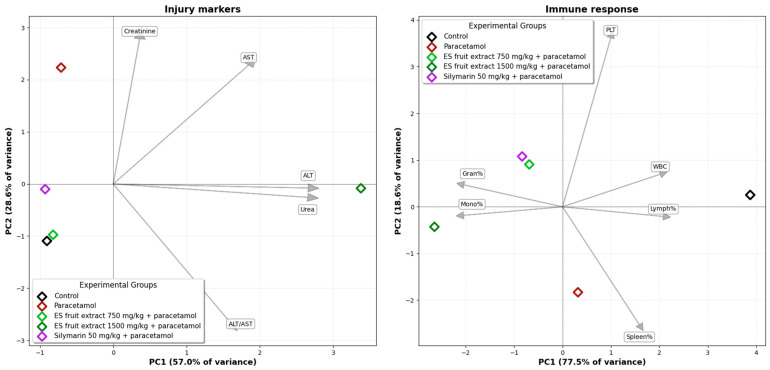
Principal Component Analysis (PCA) of the studied groups based on injury markers and on immune response parameters.

The PCA for injury markers (Figure 9) (ALT, AST, creatinine, urea) explains a total of 85.6% of the data variance across the first two axes. The group receiving the 750 mg/kg *E. senticosus* extract and the reference silymarin group are located in close proximity to the control group, indicating an effective protective action. The PCA for hematological parameters explains a total of 96.1% of the variance for the first two axes. In this analysis, the 750 mg/kg *E. senticosus* extract group clusters together with the silymarin group, indicating a similar protective effect.

The results suggest a multifaceted effect of ES, encompassing enzymatic protection, immune modulation, and antioxidant activity. The observed hematological changes, such as a decrease in leukocyte count and a shift in leukocyte populations (reduction in lymphocyte counts with an increase in monocytes and granulocytes), indicate a solid immune response induced by the toxic metabolite of paracetamol (NAPQI). Similar experiences have been previously documented [17] and interpreted as a proinflammatory and immune-damaging effect. The simultaneous enhancement of these changes by ES and silymarin may suggest an immunomodulatory effect of these substances, involving the regulation of proinflammatory cytokines and an adaptive immune response. The obtained biochemical parameters showed that ES extract at the optimal dose (750 mg/kg) stabilized liver enzyme activity (ALT, AST) and beneficially affected markers of renal function (creatinine and urea). This mechanism is likely related to the ability of ES active constituents to neutralize oxidative stress and inhibit lipid peroxidation. Similar hepatoprotective effects were also observed with silymarin, a known hepatoprotector with antioxidant properties [16].

The hepatoprotective activity observed after the administration of *E. senticosus* fruit extract at the dose of 750 mg/kg has not been fully determined, it may be associated with the antioxidant activity of the extract and it is likely to result from several complementary biological mechanisms. This mechanism could be similar to that induced by the administration of *Panax ginseng* extract, another member of the *Araliaceae* family known for its adaptogenic properties. The possible hepatoprotective effect of Black ginseng (sun-dried ginseng via repeated steaming) is primarily attributed to its antioxidant, anti-inflammatory, and antiapoptotic properties. Pretreatment with *P*. *ginseng* significantly attenuated acetaminophen-induced hepatotoxicity by restoring hepatic glutathione (GSH) levels and reducing malondialdehyde (MDA) accumulation, thereby mitigating lipid peroxidation and oxidative stress. Moreover, the extract downregulated cytochrome P450 2E1 (CYP2E1) expression, which limited the generation of reactive oxygen species (ROS) and toxic acetaminophen metabolites. In addition, *P*. *ginseng* decreased nitrative stress, as evidenced by reduced hepatic 3-nitrotyrosine (3-NT) formation, and suppressed the expression of proinflammatory enzymes, including inducible nitric oxide synthase (iNOS) and cyclooxygenase-2 (COX-2). At the cellular level, the extract modulated apoptosis-related proteins by decreasing Bax expression, increasing Bcl-2 expression, and lowering the Bax/Bcl-2 ratio, collectively contributing to reduced hepatocellular apoptosis and necrosis [24].

Given that *Eleutherococcus senticosus* also belongs to the *Araliaceae* family and exhibits comparable adaptogenic and antioxidant activities, it is plausible that its hepatoprotective mechanism may involve similar molecular pathways. The fruits contain a range of bioactive compounds, flavonoids (such as quercitin and its derivatives), phenolic acids (e.g., chlorogenic acid, dicaffeoylquinic acids), lignans, triterpenic acids (including oleanolic and ursolic acids) as well as volatile constituents [9,25,26]. These constituents have been reported to exhibit pronounced antioxidant and anti-inflammatory effects. Their activity may involve the enhancement of enzymatic antioxidant defenses, including superoxide dismutase, catalase, and glutathione peroxidase, as well as the restoration of the reduced glutathione pool and the reduction in lipid peroxidation in hepatic tissue. Su et al. [27] proved that *E. senticosus* flavonoids can significantly reduce the levels of reactive oxygen species (ROS) and malondialdehyde (MDA) in RAW cells treated with hydrogen peroxide (H_2_O_2_), thereby increasing the antioxidant capacity of cells. By improving the cellular redox balance, these compounds may protect hepatocytes from oxidative damage induced by the reactive metabolite of paracetamol. Another study showed that *E. senticosus* has a beneficial effect in treating liver injury and enhancing the antioxidant capacity, by regulating antioxidant and antiapoptotic-related gene expression level [28]. Song et al. [29] used *Edwardsiella ictaluri* infection model to demonstrate that the ES extract suppressed the expression of proinflammatory cytokines, including IL-1. The infection with *E. ictaluri* led to an upregulation of IL-1 and activation of the NF-κB/MyD88 signaling pathway, whereas treatment with the *E. senticosus* resulted in a significant downregulation of this pathway. Moreover, myo-inositol, which is also one of the components of the ES fruit extract [11], exhibits hepatoprotective activity as well. It has been proven that its administration led to an improvement in the AST/ALT ratio in patients with NAFLD, which are indicators of liver damage [30]. Myo-inositol supplementation improves liver enzymes concentrations, which is consistent with the results provided by Pan et al. [31] Zhao et al. [32] and Lee et al. [33].

Interestingly, a higher dose of ES extract (1500 mg/kg) showed a less beneficial effect, supporting the hypothesis of a paradoxical, potentially pro-oxidant effect when phytopreparations are exceeded at optimal concentrations. Available publications provide strong evidence that polyphenolic compounds and other phytoextracts—while beneficial in specific, moderate doses—can paradoxically exhibit pro-oxidant effects at higher concentrations. In this case with ES extract (1500 mg/kg) fits that dose-effect pattern, proving that at this concentration, it exhibits opposite, rather than hepatoprotective, effects. This phenomenon should be further analyzed in the context of the safe use of adaptogens, with lower but regular dosing recommended [34,35,36,37].

### 2.2. Histopathological Studies

In our previous study [11], histopathological evaluation of liver tissue revealed no pathological changes in either control animals or those treated with concentrations of *Eleutherococcus senticosus* intractum. Hepatocytes maintained normal morphology and architecture, and the overall tissue structure was preserved, indicating an absence of hepatotoxic effects. These findings are consistent with other reports confirming the safety of *E. senticosus* extracts in traditional medicine, including studies showing protective effects against liver damage induced by diabetes or heavy metal exposure [38,39].

Based on these previously established results, liver histology was not re-examined in the present study to avoid redundant publication of the same data. Instead, we focused on the brain and kidneys, as these organs represent important targets for systemic toxicity and oxidative stress. This approach allowed us to investigate potential protective effects of *E. senticosus* in other critical organs while acknowledging the previously confirmed safety of the extract in the liver.

**Figure 10:**
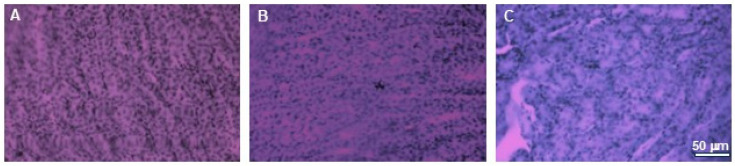
H&E staining of kidneys. Microphotographs of representative kidneys sections obtained from control mouse (**A**) and animals treated with 750 (**B**) or with 1500 mg/kg b.w. of the intractum (**C**). See the proper morphology and cell arrangement within all the sections. Magnification: 400×, bar 50 μm.

To analyze the effect on other tissues in the body, we examined the effect of extracts supply on the induction of cytotoxic effects in organs such as the kidneys and brains. Histopathological examination did not reveal any pathological changes in those organs obtained from mice in any experimental group. Minor changes to the kidneys were observed in one mouse in the group treated with 750 mg/kg b.w. of the intractum. Areas containing cells with irregular morphology and slight tubule degeneration were present. This animal also exhibited areas of lightening in H&E staining of the liver. However, the liver cells displayed normal morphology and arrangement [11]. The other animals in the study group did not show any changes indicating kidney damage. Their kidneys showed proper cell morphology and architecture (Figure 10).

**Figure 11:**
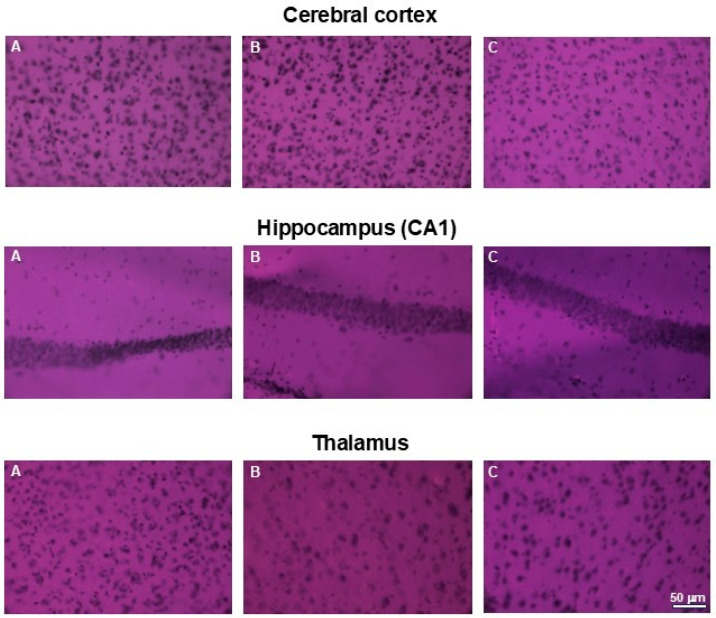
H&E staining of brains. Microphotographs of representative brain sections obtained from control mouse (**A**) and animals treated with 750 (**B**) or with 1500 mg/kg b.w. of the intractum (**C**). **Upper** panel shows cerebral cortex, **middle** and **bottom** panels show selected subcortical structures: CA1 region of hippocampus and thalamus (respectively). Note the proper cell arrangement and morphology within all the sections. Magnification: 400×, bar 50 mm.

Similarly, no abnormalities in cell morphology or arrangement were observed in the brains of the examined animals. Figure 11. presents representative areas of the cerebral cortex and subcortical regions from animals in the control and both treated groups.

There were no reports of histopathological changes in the brain or kidneys caused by the administration of *Eleutherococcus senticosus*. In a study in rats with a cerebral ischemia model (4-VO), doses of 3, 30, and 300 mg/kg of ES ethanol bark extract protected hippocampal cells (CA1), reduced apoptosis, and triggered a beneficial immunohistochemical effect (reduction in COX-2, GFAP, and OX-42 antibody)—meaning no pathological changes. Immunohistochemical findings suggest that this effect may be linked to its anti-inflammatory activity, achieved through the suppression of COX-2, as well as reduced activation of microglia and astrocytes [40]. No histopathological changes were observed; on the contrary, a neuroprotective effect was observed.

Adaptogens as a group of medicinal plants, should demonstrate low levels of cytotoxicity in tissues, even with long-term use. For instance, the safety impact of long-term *Panax ginseng* supplementation was examined. No negative changes were observed during the 12-month treatment period in rat experiment model, no significant organ changes were observed, histopathological, hematological and serum biochemical analyses did not show any significance modifications proving the safety of using this raw material from the adaptogenic group [41].

### 2.3. Phytochemical Characterization-Amino Acid Profile

**Figure 12:**
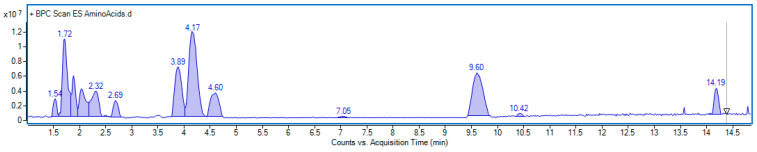
Base peak chromatogram obtained in positive ionization mode of *E. senticosus* fruit extract analyzed for amino acid content. Identified compounds are listed in Table 1.

Our study showed that the amino acid profile of *E. senticosus* (ES) fruit extract was diverse and included twenty components (Figure 12).

Ten compounds were identified based on their characteristic mass spectra, while the remaining ten were detected through extracted ion chromatograms within specific mass ranges. Quantitative analysis revealed that tyrosine, leucine, phenylalanine, and isoleucine were the predominant amino acids, with concentrations of 3.98, 3.55, 2.75, and 2.13 mg/g of dry weight, respectively. Furthermore, moderate amounts of serine, glutamic acid, arginine, tryptophan, valine, and norvaline were also detected, ranging from 1.05 to 1.54 mg/g. The detailed results of the quantification are summarized in Table 2. Examples of mass spectra of main amino acids and extracted ion chromatograms used for quantification are included in the Supplementary Material (Figures S1 and S2).

Amino acids play a crucial role in hepatoprotective actions, supporting liver health and function. They are essential for the synthesis of proteins, including enzymes and detoxifying agents that aid in liver cell regeneration and the neutralization of toxins. Specific amino acids, such as glycine, glutamate, methionine and cysteine, contribute to the production of glutathione–a key antioxidant that protects liver cells from oxidative stress [42]. Thus, adequate dietary intake of amino acids can support detoxification processes and promote hepatic recovery.

### 2.4. Low Molecular Weight Organic Acid

Three low molecular weight organic acids were detected in the extract in significant amounts, including citric, malic, and gluconic acid (Figure S3). Among them, malic acid was predominant, followed by citric acid with the content of 25.1 and 8.4 µg/g, respectively. The results of the quantification are presented in Table 3.

Low molecular weight organic acids, such as citric and malic acid, may play a supportive role in liver function through several biochemical mechanisms. As intermediates of the tricarboxylic acid (TCA) cycle, they contribute to cellular energy production, which is essential for the liver’s metabolic and detoxifying activities. Additionally, citric acid exhibits antioxidant and metal-chelating properties, potentially reducing oxidative stress in hepatic tissue [43,44].

### 2.5. Secondary Metabolites

**Figure 13:**
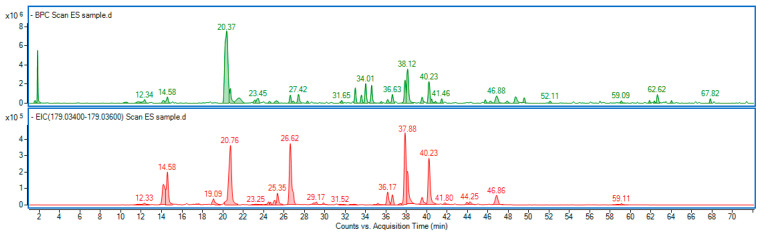
Base peak chromatogram (green line) of *Eleutherococcus senticosus* fruit extract, along with the extracted ion chromatogram (red line), showing a mass range corresponding to caffeic acid (C_9_H_8_O_4_) a ppm error of −5.46 to 5.65. Identified compounds are listed in Table S1.

Our study showed that the ES extract is rich in phenolic compounds, particularly derivatives of caffeic acid, namely caffeoylquinic acids and dicaffeoylquinic acids (Figure 13). Among them, 3-O-caffeoylquinic acid (common name: chlorogenic acid) was predominant. The detailed chromatographic and mass spectrometric data of identified phenolic constituents are included in the Supplementary Material Table S1.

The analysis revealed a diverse profile of phenolic compounds, with phenolic acids being predominant. Among the phenolic acids, chlorogenic acids were the most abundant, with a content of 28.02 ± 1.21 mg/g, followed by dicaffeoylquinic acids (13.42 ± 0.76 mg/g). Other identified compounds included caffeoylshikimic acids, feruloylquinic acids, and protocatechuic acid. The total phenolic acid content was 43.77 mg/g. In contrast, flavonoid content was significantly lower, amounting to 2.22 mg/g. Among the flavonoids, quercetin 3-O-hexosides, including glucoside and galactoside forms, as well as quercetin O-rutinosides, were the most prominent. Catechin and its hexoside derivative were detected in smaller amounts. Free quercetin was also identified, with a content of 0.19 ± 0.01 mg/g. The results of phenolic acid and flavonoids quantification expressed as total amount of different isomeric forms are summarized in Table 4.

Anthocyanins in the ES extract were represented by one dominant constituent (2.79 ± 0.14 mg/g) at retention time 21.55 min, characterized by a typical UV-Vis absorption spectrum with a maximum in the range of 520–530 nm. The compound exhibited [*m*/*z*+H]^+^ = 581.15154 (estimated formula: C_26_H_28_O_15_, ppm error: 2.49), with a characteristic fragment ion at [*m*/*z*+H] = 287.05548 (estimated formula: C_15_H_10_O_6_, ppm error: 1.63), corresponding to the neutral aglycone form of cyanidin (Figure S4).

Furthermore, based on literature data regarding characteristic constituents of *Eleutherococcus* species [45], additional secondary metabolites were investigated based on extracted ion approach. Quinic acid (cyclohexanecarboxylic acid, formula: C_7_H_12_O_6_) was extracted within [*m*/*z*−H] range 191.055–191.057 (a mass error from −5.76 to 4.63 ppm) and it was found in significant amount of 11.56 ± 0.87 mg/g. Eleutheroside E (syringaresinol) belonging to the lignan family, identified by the characteristic adduct [*m*/*z*+HCOOH]^−^ = 787.26391 and parent ion [*m*/*z*+H]^−^ = 741.26173 (estimated formula: C_34_H_46_O_18_, mass error: 2.32 and 0.8 ppm, respectively) was detected at a concentration of 0.22 ± 0.02 mg/g. Triterpenic acids were also detected based on extracted ions corresponding to the mass range characteristic for these compounds ([M–H]^−^ = 455.350–455.355). The analysis revealed two peaks: one corresponding to a mixture of oleanolic and ursolic acid (total content: 7.24 µg/g of extract), and another representing an unidentified triterpenic acid (content: 14.32 µg/g). Extracted ion chromatograms of mentioned compound are presented in Figure S5.

Secondary metabolites are organic compounds produced by plants that are not directly involved in their growth or development. They are classified as non-nutritional food components, as they are not essential nutrients like carbohydrates, proteins, or fats. However, they are important for human health and nutrition due to their wide range of biological activities. Among secondary metabolites, phenolic compounds—such as phenolic acids and flavonoids—are of particular interest. These compounds have been shown to exhibit strong antioxidant, anti-inflammatory, and antimicrobial properties. They help protect the body from oxidative stress, modulate enzyme activity, and influence signaling pathways involved in chronic diseases such as cardiovascular disorders, diabetes, and certain types of cancer. Therefore, their presence in the diet contributes significantly to the promotion of health and the prevention of disease.

### 2.6. Mineral Composition

The elemental composition of the extract is presented in Table 5. Among the analyzed elements, potassium (K) was the most abundant, with a concentration of 24,181 µg/g and a coefficient of variation (CV) of 0.27%, followed by calcium (Ca) at 5788 µg/g and magnesium (Mg) at 1369 µg/g. Sodium (Na) and selenium (Se) were also present in significant amounts, at 291.7 µg/g and 292.9 µg/g, respectively, both with very low variability (CVs of 0.16% and 0.09%). Zinc (Zn) was detected at a lower concentration of 33.12 µg/g, with a higher CV of 3.15%, indicating slightly greater variability in measurements. Iron (Fe), copper (Cu), and manganese (Mn) were not detected (nd) under the applied analytical conditions, suggesting their concentrations were below the detection limits of the method. Example of a spectral window for the analysis of potassium, magnesium, and zinc using inductively coupled plasma optical emission spectrometry (ICP-OES) is presented in Supplementary Material (Figure S6).

Trace elements are fundamental for liver function and protection [46]. For example, zinc is a cofactor for over 300 enzymes. It stabilizes cell membranes, regulates immune responses, and supports liver regeneration and detoxification. Zinc deficiency is common in liver diseases like cirrhosis and fatty liver and is linked to impaired immune function and slower hepatocyte repair. Magnesium is as a cofactor in ATP synthesis and metabolic regulation. It also has anti-inflammatory effects and aids in detox processes. Disruptions in magnesium homeostasis are observed in cirrhosis and have been associated with worse liver outcomes. Selenium is integral to selenoproteins like glutathione peroxidase, which combats lipid peroxidation and oxidative stress. Patients with chronic liver conditions often exhibit low selenium levels, while supplementation has shown benefits in non-alcoholic fatty liver disease (NAFLD) by reducing oxidative damage and inflammation. However, it should be noted that the roles of some elements, such as iron and copper, are dual. On one hand, they are essential for metabolic activity; on the other, when present in excess, they may accumulate in hepatocytes and catalyze oxidative damage.

## 3. Materials and Methods

### 3.1. Plant Material

The extract was prepared from the fruits of *E. senticosus* (Rupr. et Maxim.) Maxim., following the procedures described in our previous work [47]. The fruits were collected from the Medicinal and Cosmetic Plant Garden in Bydgoszcz, Poland, in September 2024 (53°07′36.55″ N; 18°01′51.64″ E). Plant samples were deposited at the Department of Pharmaceutical Botany and Pharmacognosy of the Collegium Medicum in Bydgoszcz (Cat. Nr. ES/9/2024). The authenticity of the plant material was verified with morphological examination and confirmed by HPLC-DAD analysis, comparing the results with reference standards. Briefly, the process of extraction involved macerating fresh fruits (20 g) in 100 mL of 40% (*v*/*v*) ethanol (Cat. No.: E9508, Merck KGaA, Darmstadt, Germany) for 30 days at room temperature in the absence of light (this type of preparation is referred to as *intractum*). After maceration, the mixture was filtered through Whatman No. 4 filter paper. The solvent was then removed under reduced pressure at 45 °C, and the residue was frozen at −20 °C and subsequently lyophilized. The resulting lyophilized extract was stored at 4 °C until further use.

### 3.2. Evaluation of the Hepatoprotective Effect of the Eleutherococcus senticosus Fruit Extract in a Model of Liver Damage with Paracetamol

A detailed study protocol, including the research question, key design features, and analysis plan, was prepared prior to the experiment in accordance with local ethical committee requirements, but it was not registered in a public database.

The female BALB/c mice (*n* = 25, 8 weeks old) were used in the study. Animals were housed under specific pathogen-free (SPF) conditions in individually ventilated cages (5 animals per cage) at 21–23 °C, with 50% relative humidity and a 12 h light/dark cycle. Standard laboratory chow and water were provided ad libitum. All experimental procedures were approved by the Local Ethics Committee (approval number: 055/2024/P1). After a quarantine period, mice were randomized into five groups (*n* = 5 per group) based on body weight:

- Group 1 (Control): Water (per os (PO), 0.1 mL/10 g) for 7 days + vehicle (EtOH/PEG/NaCl) intraperitoneal (IP) on day 8,
- Group 2: Paracetamol (300 mg/kg, IP, day 8),
- Group 3: *Eleutherococcus senticosus* fruit extract (750 mg/kg, PO, twice daily for 7 days and once on day 8) + paracetamol (300 mg/kg, IP, day 8),
- Group 4: *Eleutherococcus senticosus* fruit extract (1500 mg/kg, PO, twice daily for 7 days and once on day 8) + paracetamol (300 mg/kg, IP, day 8),
- Group 5: Silymarin (50 mg/kg, PO, twice daily for 7 days and once on day 8) + paracetamol (300 mg/kg, IP, day 8).

PO administrations were performed using a flexible gastric gavage tube. Paracetamol was dissolved in a mixture of 96% EtOH, PEG, and 0.9% NaCl (5:10:85, *v*/*v*/*v*) (Pol-Aura, Warsaw, Poland; EtOH cat. no.: PA-06-396420113; PEG cat. no.: PA-04-F1243; NaCl cat no.: PA-31-HI7037L) due to low water solubility. Silymarin (Silimax, Filofarm, Bydgoszcz, Poland) was prepared as a water suspension. The experimenter, who was responsible for conducting the study, was aware of the group allocation throughout the experimental procedure. All animals were included in the experiment and analysis, and no exclusion criteria were applied; all data points were taken into account according to generally accepted standards.

Twenty-four hours after paracetamol administration (day 9), mice were euthanized, following analgesia with buprenorphine and anesthesia with isoflurane; blood and organs (liver, kidneys, spleen, lungs, brain) were collected. Blood was used for hematological and biochemical analyses (ALT, AST, urea, creatinine, total protein, albumin) and serum isolation. Organs were weighed and fixed in 10% neutral-buffered formalin for histopathological evaluation.

### 3.3. Histopathological Studies

The brains and kidneys of animals from all experimental groups were examined to assess cell morphology and arrangement of individual structures. Histological marking was performed using hematoxylin and eosin (H&E) staining. Fixed biological material from each experimental group was cryoprotected by immersion in 10, 20 and 30% (*w*/*v*) sucrose solutions in PBS (Merck KGaA, Darmstadt, Germany), cat. no.: P3813 (for 24 h, 3 days and 6 days, respectively). Next, the brains and kidneys were frozen on dry ice, sectioned into 20-μm thick slices using a CM 1850 UV cryostat (Leica, Wetzlar, Germany). The sections were processed for histological/morphological analysis using routine hematoxylin and eosin staining (hematoxylin No. 1.50174 and eosin No. 1.09844, Merck, Darmstadt, Germany). Finally, the stained sections were mounted with DPX mounting medium (Sigma-Aldrich, Schnelldorf, Germany), cat. no.: 06522. Images were examined and captured using a Nikon light microscope (Nikon, Tokyo, Japan) equipped with a CCD camera and image analysis system.

### 3.4. Ultra-High Performance Liquid Chromatography (UHPLC-DAD-MS)

Ultra-high-performance liquid chromatograph (UHPLC) Infinity Series II with a DAD detector and an Agilent 6224 ESI/TOF mass spectrometer (Agilent Technologies, Santa Clara, CA, USA) coupled with a Kinetex C18 column 1.7 µm, 150 mm × 2.1 mm (Phenomenex, Torrance, CA, USA) was used in the investigation. The column temperature was 30 °C. Water with 0.05% formic acid (LiChropur^®^, LC–MS grade; Merck KGaA, Darmstadt, Germany), cat. no. 113228 (solvent A) and acetonitrile LC–MS, LiChrosolv^®^ hypergrade (Merck KGaA, Darmstadt, Germany), cat. no. 100029 with 0.05% formic acid (solvent B) at the flow rate 0.2 mL/min was used as a mobile phase. Phenolic compounds were separated according to following elution gradient: 0–2 min from 98% A, 2–35 min from 98% A to 80% A, 35–50 min from 80% A to 75% A, 50–60 min from 75% A to 60% A, 60–85 min from 60% A to 35% A. Triterpenic acids were analyzed using 25% solvent A and 75% solvent B. Amino acids and low molecular weight organic acids were analyzed according to following elution gradient 0–5 min 100% A and 5–20 min from 100% A to 75% A. LC–MS conditions: the drying gas temperature was set at 325 °C, with a gas flow rate of 8 L/min. The nebulizer pressure was maintained at 30 psi, while the capillary voltage was set to 3500 V. The skimmer voltage was 65 V, and the fragmentor voltage was 140 V and 240 V in negative mode and 120 V in positive mode.

### 3.5. Elemental Analysis

Samples of the extract were digested using a mixture of nitric acid (HNO_3_) Suprapur^®^ (Merck KGaA, Darmstadt, Germany), cat. no. 100441 and distilled water in a volume ratio of 2:8 (*v*/*v*). The digestion was carried out with a DigiPREP MS heating block (SCP Science, Canada), following standard procedures to ensure complete decomposition of organic matter. Elemental concentrations were determined using inductively coupled plasma optical emission spectrometry (ICP-OES) with a PlasmaQuant PQ 9000 Elite instrument (Analytik Jena AG, Jena, Germany), as previously described [48]. The following operating conditions were used for the analysis: RF Power: 1300 W; Plasma Gas Flow Rate: 12.0 L/min, Auxiliary Gas Flow Rate: 0.50 L/min, Nebulizer Gas Flow Rate: 0.60 L/min, Observation Mode: Axial direction (with attenuated axial mode used for Na and K), Readout Time: 3 s (6 s for selenium). Outliers in the dataset were identified using Grubbs’ test at a 95.4% confidence level (corresponding to 2 sigma). Calibration was carried out using single-element standards PlasmaCAL, ISO 17034/ISO 17294 (SCP Science, Baie-D’Urfé, QC, Canada) series (1000 ppm), appropriately diluted. Calibration verification was performed using a multi-element standard PlasmaCAL, ISO 17034/ISO 17294 (SCP Science, Baie-D’Urfé, QC, Canada) at two concentration levels: 200 ppb and 400 ppb, with recovery rates within the range of 95–105% [49,50].

### 3.6. Statistical Analysis

Data were analyzed using GraphPad Prism 7. Non-parametric Kruskal–Wallis test was used to compare treatment groups with the control group. A *p*-value < 0.05 was considered statistically significant. To investigate the complex relationships between the experimental groups and the measured parameters, multivariate data analyses were performed. Principal Component Analysis (PCA) was conducted to visualize the interrelationships between samples and variables. Three separate PCA models were generated:

- on the complete dataset, including all biochemical and hematological parameters,
- on a subset of data containing only injury markers (ALT, AST, creatinine, urea),
- on a subset of data comprising hematological and immune response parameters.

The results were presented as biplots, displaying the scores for individual samples and the vectors for each variable. Principal Component Analyses were performed using Python 3.13. The Hierarchical Cluster Analysis was conducted and the dendrogram was generated using Statistica 13.3 software. The stability of the clusters identified by HCA was assessed using bootstrap validation with 1000 iterations.

## 4. Conclusions

Although *Eleutherococcus senticosus* fruits are growing in popularity as food ingredients, rigorous evidence-based safety is still needed. The present study provides new evidence supporting the hepatoprotective potential of *Eleutherococcus senticosus* fruit extract in a mouse model of paracetamol-induced liver injury. Pre-treatment with the extract, particularly at a dose of 750 mg/kg, helped maintain liver enzyme activity (ALT, AST) and renal function markers closer to physiological levels compared to the paracetamol-only group, while modulating immune parameters and platelet indices. No histopathological changes were observed in the kidneys or brains of treated animals, confirming the safety of the extract at the tested doses. However, the higher dose (1500 mg/kg) demonstrated reduced benefits of usage, in some biochemical parameters, suggesting a possible paradoxical effect associated with excessive intake of plant-based preparations. Further studies are needed to clarify dose-dependent mechanisms and establish safe and effective dosing regimens for adaptogens in hepatoprotection.

## Figures and Tables

**Table 1:** Organ mass per body mass [%].

Group	% Body Mass				
	Liver	Kidneys	Spleen	Lungs	Brain
1. control	4.43	1.19	0.44	0.64	2.09
2. paracetamol 300 mg/kg	4.45	1.28	0.44	0.76	2.23
3. ES fruit extract 750 mg/kg + paracetamol	4.20	1.3	0.41	0.74	2.27
4. ES fruit extract 1500 mg/kg + paracetamol	4.25	1.28	0.4	0.73	2.37
5. Silymarin 50 mg/kg + paracetamol	4.19	1.31	0.39	0.74	2.21

**Table 2:** Results of quantification the amino acids identified in *E. senticosus* fruit extract.

R_T_ (min.)	Extraction Window [*m*/*z*+H]	Mass Error ppm Range	Formula	Compound	Content (mg/g ± SD)
1.57	147.112–147.114	−5.5 to 8.18	C_6_H_14_N_2_O_2_	lysine	0.36 ± 0.02
1.69	156.076–156.078	−4.86 to 8.04	C_6_H_9_N_3_O_2_	histidine	0.09 ± 0.01
1.69	175.118–175.12	−5.47 to 6.02	C_6_H_14_N_4_O_2_	arginine	1.15 ± 0.07
1.69	76.039–76.04	−4.06 to 9.26	C_2_H_5_NO_2_	glycine	0.27 ± 0.02
1.70	106.049–106.051	−8.28 to 10.76	C_3_H_7_NO_3_	serine	1.54 ± 0.11
1.72	134.044–134.046	−5.89 to 9.14	C_4_H_7_NO_4_	aspartic acid	0.08 ± 0.01
1.73	120.065–120.067	−4.36 to 12.43	C_4_H_9_NO_3_	threonine	1.01 ± 0.08
1.72	90.054–90.056	−10.72 to 11.54	C_3_H_7_NO_2_	alanine	0.98 ± 0.06
1.74	147.075–147.077	−9.71 to 3.98	C_5_H_10_N_2_O_3_	glutamine	0.69 ± 0.04
1.77	148.059–148.061	−9.75 to 3.85	C_5_H_9_NO_4_	glutamic acid	1.36 ± 0.09
1.89	116.07–116.072	−5.26 to 12.12	C_5_H_9_NO_2_	proline	0.63 ± 0.04
2.05	118.085–118.087	−10.72 to 6.36	C_5_H_11_NO_2_	valine	1.25 ± 0.07
2.24	241.03–241.033	−4.69 to 7.81	C_6_H_12_N_2_O_4_S_2_	cystine	detected
2.30	118.085–118.0087	−10.72 to 6.36	C_5_H_11_NO_2_	norvaline	1.13 ± 0.08
2.69	150.057–150.059	−8.89 to 4.52	C_5_H_11_NO_2_S	methionine	0.42 ± 0.31
3.87	132.101–132.103	−6.9 to 8.35	C_6_H_13_NO_2_	isoleucine	2.13 ± 0.17
4.17	132.101–132.103	−6.9 to 8.35	C_6_H_13_NO_2_	leucine	3.55 ± 0.24
4.60	182.080–182.082	−6.46 to 4.59	C_9_H_11_NO_3_	tyrosine	3.98 ± 0.21
9.61	166.085–166.087	−7.6 to 4.51	C_9_H_11_NO_2_	phenylalanine	2.75 ± 0.11
14.19	205.096–205.098	−5.65 to 4.14	C_11_H_12_N_2_O_2_	tryptophan	1.05 ± 0.07
			**Total amino acids**		**24.42**

**Table 3:** Results of quantification of low molecular organic acid identified in *E. senticosus* fruit extract.

R_T_ (min)	Observed Ion Mass [M–H]^−^	Mass Error (ppm)	Formula	Identified	Content (µg/g ± SD)
1.77	195.05153	2.57	C_6_H_12_O_7_	gluconic acid	2.11 ± 0.14
2.29	133.01489	4.80	C_4_H_6_O_5_	malic acid	25.12 ± 1.14
3.39	191.01882	−4.72	C_6_H_8_O_7_	citric acid	8.39 ± 0.36
				**Total acids**	**61.46**

**Table 4:** The results of quantification of identified phenolic acids and flavonoids found in *E. senticosus* fruit extract expressed as milligrams per gram of dried extract.

Observed Ion Mass [*m*/*z*−H]^−^	Formula	Identified	Content (mg/g ± SD)
153.01932	C_7_H_6_O_4_	Protocatechuic acid	0.43 ± 0.02
353.087–353.089	C_16_H_18_O_9_	Chlorogenic acids	28.02 ± 1.21
335.077–335.079	C_16_H_16_O_8_	Caffeoylshikimic acids	1.03 ± 0.05
367.103–367.104	C_17_H_20_O9	Feruloylquinic acids	0.45 ± 0.02
515.118–515.121	C_25_H_24_O_12_	Dicaffeoylquinic acids	13.42 ± 0.76
207.06715	C_11_H_12_O_4_	Caffeic acid derivative	0.42 ± 0.02
		**Total phenolic acids**	**43.77**
451.12473	C_21_H_24_O_11_	Catechin hexoside	0.13 ± 0.01
289.07182	C_15_H_14_O_6_	Catechin	0.28 ± 0.02
609.145–609.148	C_27_H_30_O_16_	Quercetin O-rutinosides	0.74 ± 0.02
463.088–463.09 (300)	C_21_H_20_O_12_	Quercetin 3-O-hexosides	0.88 ± 0.03
301.03656	C_15_H_10_O_7_	Quercetin	0.19 ± 0.01
		**Total flavonoids**	**2.22**

**Table 5:** Elemental composition of the *E. senticosus* fruit extract determined by ICP-OES.

Element	Wavelength (nm)	Content (µg/g)	CV (%)
Ca	315.8869	5788	0.08
K	766.4911	24,181	0.27
Mg	258.2162	1369	0.69
Na	589.5924	291.7	0.16
Se	196.0280	292.9	0.09
Zn	206.2000	33.12	3.15
Fe	259.9396	nd	–
Cu	327.3960	nd	–
Mn	257.6100	nd	–

nd—not detected.

## Data Availability

The original contributions presented in this study are included in the article/Supplementary Material. Further inquiries can be directed to the corresponding author.

## Supplementary Materials

The following supporting information can be downloaded at https://www.mdpi.com/article/10.3390/nu17213456/s1, Table S1: Chromatographic and mass data of phenolic constituents identified in *Eleutherococcus senticosus* fruit extract. Figure S1: Mass spectrum of the main amino acids found in *E. senticosus* fruit extract including tyrosine (R_t_ = 4.60), phenylalanine (R_t_ = 9.60), leucine (R_t_ = 4.17), and tryptophan (R_t_ = 14.19); Figure S2: Example of extracted ion chromatograms showing spectral window characteristic for proline, valine/norvaline, methionine, glutamine, and glutamic acid; Figure S3: Base peak chromatogram in negative mode showing analysis of low molecular organic acids including gluconic acid (R_T_ = 1.77 min), malic acid (R_T_ = 2.29 min), and citric acid (R_T_ = 3.39); Figure S4: UV-Vis spectrum and mass spectrum of the anthocyanin found in *E. senticosus* fruit extract; Figure S5: Extracted ion chromatogram from *E. senticosus* fruit extract (blue line) and standard (red line), showing a extraction window with mass range corresponding to quinic acid (C_7_H_12_O_6_, a mass error from −5.76 to 4.63 ppm), Eleutheroside E (C_34_H_46_O_18_, a mass error from −4.87 to 4.56), and ursolic acid (C_30_H_48_O_3_, a mass error from −6.72 to 4.23); Figure S6: Example of a spectral window for the analysis of potassium, magnesium, and zinc using inductively coupled plasma optical emission spectrometry (ICP-OES).

## Abbreviations

ALT	alanine aminotransferase
AST	aspartate aminotransferase
ATP	adenosine triphosphate
COX-2	cyclooxygenase-2
CV	coefficient of variation
ES	*Eleutherococcus senticosus*
EtOH/PEG/NaCl	ethanol/polyethylene glycol/sodium chloride
GFAP	glial fibrillary acidic protein
H&E	hematoxylin and eosin
HGB	hemoglobin
HPLC-DAD	high-performance liquid chromatography with diode-array detection
ICP-OES	inductively coupled plasma optical emission spectrometry
IP	intraperitoneal
MVP	mean platelet volume
NAFLD	non-alcoholic fatty liver disease
NAPQI	*N*-acetyl-p-benzoquinoneimine
PCT	plateletcrit
PDW	platelet distribution width (platelet anisocytosis)
PO	per os (by mouth)
RBC	red blood cells
TCA	tricarboxylic acid
UHPLC	ultra-high-performance liquid chromatography
