# Supplementary material for: Hepatoprotective Activity of the Fruits of Eleutherococcus senticosus in Acetaminophen-Induced Liver Injury in Mice and Their Chemical Composition
Source: Nutrients. 2025 Nov 1;17(21):3456. doi: 10.3390/nu17213456 (PMC12610257; doi:10.3390/nu17213456)
Supplement: Supplementary file 1 [file nutrients-17-03456-s001.zip › nutrients-3941127-supplementary.pdf]

nutrients

MDPI

Table S1. Chromatographic and mass data of phenolic constituents identified in *Eleutherococcus senticosus* fruit extract.

Rt (min)	Observed ion mass [M-H]⁻ (characteristic fragments)	Δ ppm	Formula	Identified
1.83	191.05691	4.16	$C_7H_{12}O_6$	Quininc acid
10.33	315.07238	0.71	$C_{13}H_{16}O_9$	Protocatechuoylglucose
10.58	153.01947 (109)	0.89	$C_7H_6O_4$	Protocatechuic acid*
14.58	353.08877 (191,179)	2.72	$C_{16}H_{18}O_9$	Neochlorogenic acid*
18.47	451.12491 (289)	0.72	$C_{21}H_{24}O_{11}$	Catechin hexoside
18.77	289.07201	0.86	$C_{15}H_{14}O_6$	Catechin*
20.37	353.08839 (191,179)	1.65	$C_{16}H_{18}O_9$	Chlorogenic acid*
23.45	353.08852 (191,179)	2.02	$C_{16}H_{18}O_9$	Cryptochlorogenic*
24.65	335.07789 (179)	1.93	$C_{16}H_{16}O_8$	Caffeoylshikimic acid
25.32	367.10379 (191,173)	0.91	$C_{17}H_{20}O_9$	Feruloylquinic acid
25.34	335.07791 (179)	1.99	$C_{16}H_{16}O_8$	Caffeoylshikimic acid
26.62	335.07802 (179)	2.32	$C_{16}H_{16}O_8$	Caffeoylshikimic acid
27.55	367.10398 (191,173)	1.42	$C_{17}H_{20}O_9$	Feruloylquinic acid
33.01	609.14729	1.94	$C_{27}H_{30}O_{16}$	Quercetin 7-O-rutinoside*
33.60	609.14691(463,300)	1.31	$C_{27}H_{30}O_{16}$	Quercetin 3-O-rutinoside*
34.01	463.08892 (300)	1.55	$C_{21}H_{20}O_{12}$	Quercetin 3-O-galactoside*
34.62	463.08801 (300)	-0.41	$C_{21}H_{20}O_{12}$	Quercetin 3-O-glucoside*
36.17	515.11966 (353,191,179)	0.31	$C_{25}H_{24}O_{12}$	Dicaffeoylquinic acid
36.63	515.11949 (353,191,179)	-0.02	$C_{25}H_{24}O_{12}$	Dicaffeoylquinic acid
37.93	515.12018 (353,191,179)	1.32	$C_{25}H_{24}O_{12}$	3,5 – Dicaffeoylquinic acid*
38.12	515.12045 (353,191,179)	1.84	$C_{25}H_{24}O_{12}$	Dicaffeoylquinic acid
40.21	515.12017 (353,191,179)	1.30	$C_{25}H_{24}O_{12}$	4,5-Dicaffeoylquinic acid*
46.88	207.06701 (179,161,133)	3.50	$C_{11}H_{12}O_4$	Caffeic acid derivative
48.73	301.03621	2.76	$C_{15}H_{10}O_7$	Quercetin*

* Confirmed using standards

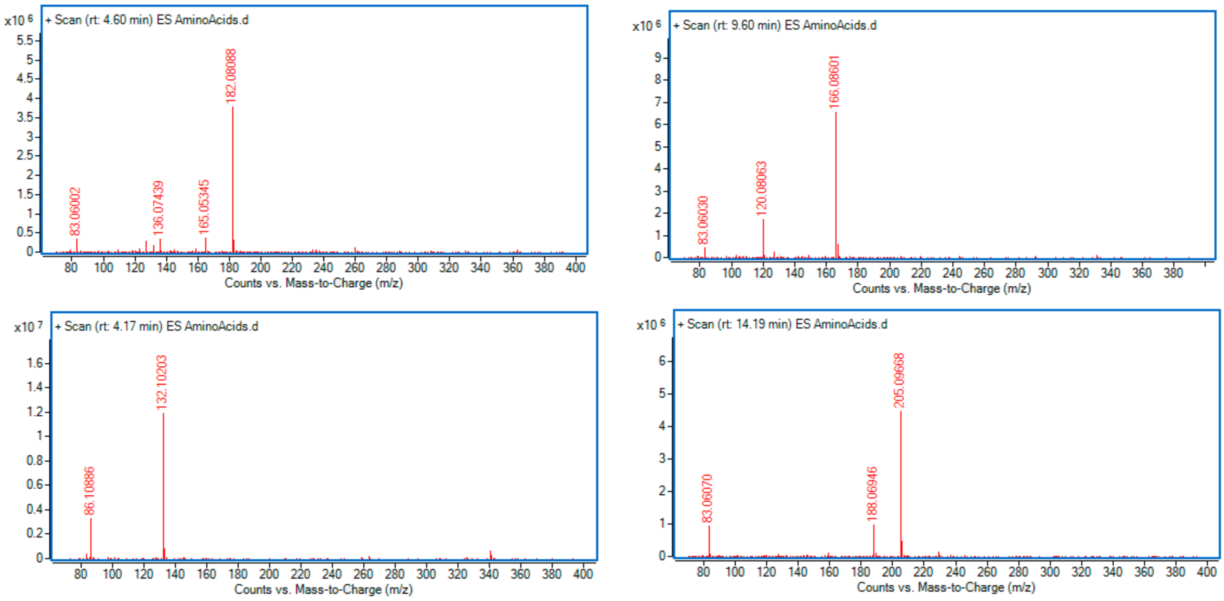

Figure S1. Mass spectrum of the main amino acids found in *E. senticosus* fruit extract including tyrosine (R_t=4.60), phenylalanine (R_t=9.60), leucine (R_t=4.17), and tryptophan (R_t=14.19).

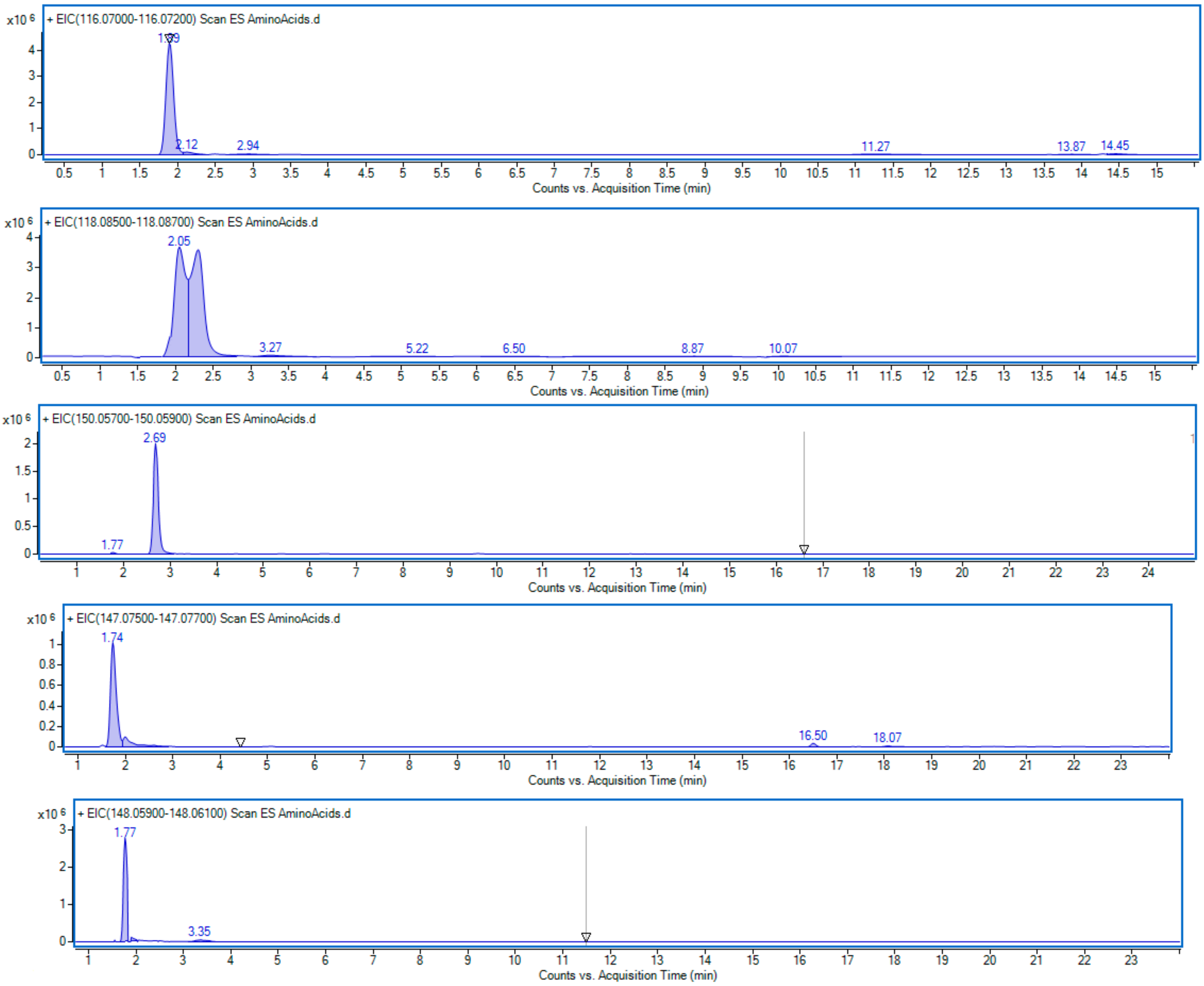

Figure S2. Example of extracted ion chromatograms showing spectral window characteristic for proline, valine/norvaline, methionine, glutamine, and glutamic acid

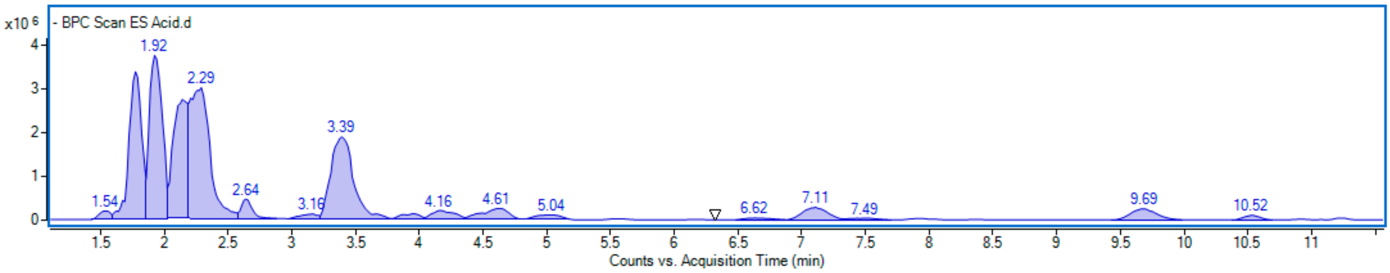

Figure S3. Base peak chromatogram in negative mode showing analysis of low molecular organic acids including gluconic acid (R_T=1.77 min), malic acid (R_T=2.29 min.), and citric acid (R_T=3.39).

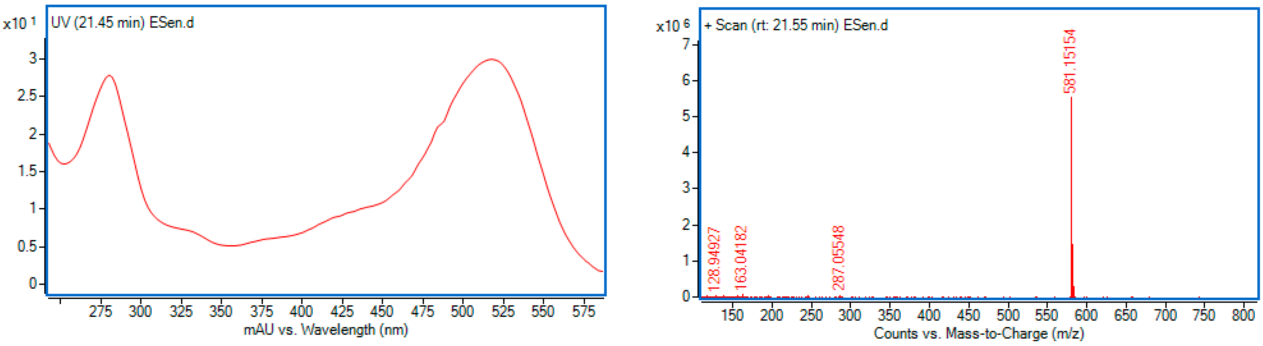

Figure S4. UV-Vis spectrum and mass spectrum of the anthocyanin found in *E. senticosus* fruit extract

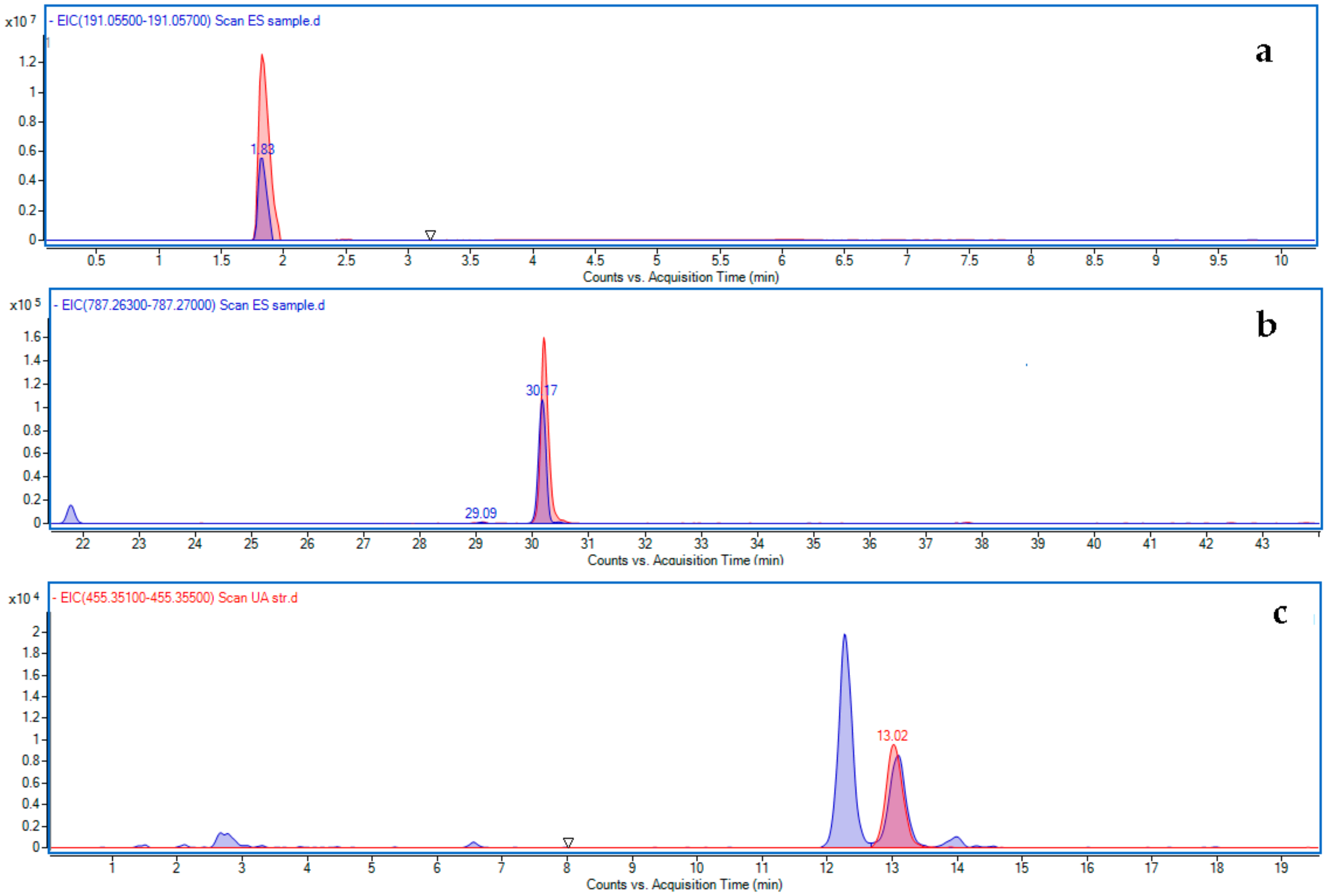

Figure S5. Extracted ion chromatogram from *E. senticosus* fruit extract (blue line) and standard (red line), showing a extraction window with mass range corresponding to quinic acid ($C_7H_{12}O_6$, a mass error from -5.76 to 4.63 ppm), Eleutheroside E ($C_{34}H_{46}O_{18}$, a mass error from -4.87 to 4.56), and ursolic acid ($C_{30}H_{48}O_3$, a mass error from -6.72 to 4.23)

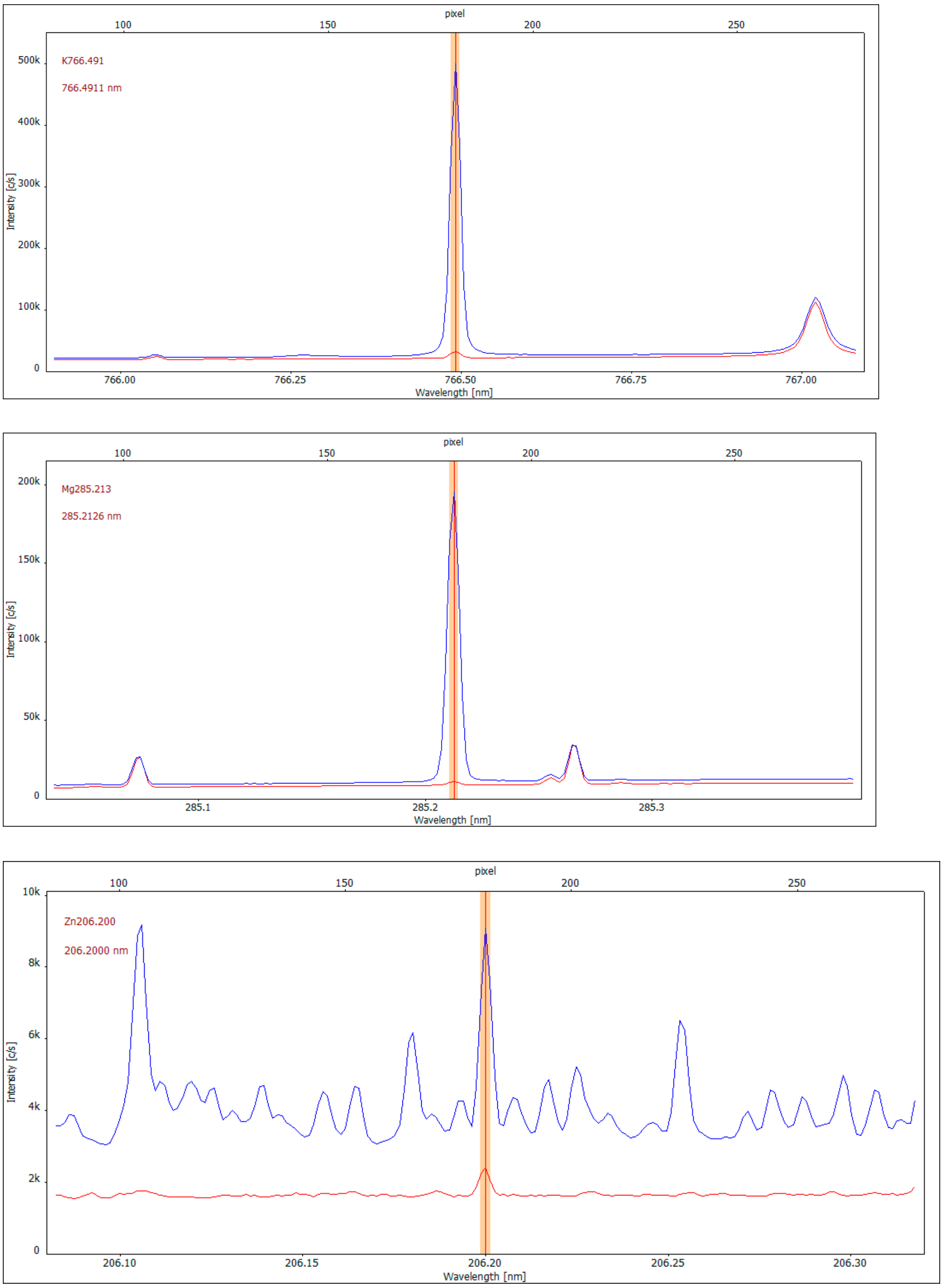

Figure S6. Example of a spectral window for the analysis of potassium, magnesium, and zinc using inductively coupled plasma optical emission spectrometry (ICP-OES).
